# Red blood cell triglycerides—a unique pool that incorporates plasma-free fatty acids and relates to metabolic health

**DOI:** 10.1016/j.jlr.2021.100131

**Published:** 2021-10-04

**Authors:** Yilin Song, Michael D. Jensen

**Affiliations:** Division of Endocrinology, Diabetes and Metabolism, Endocrine Research Unit, Mayo Clinic, Rochester, MN, USA

**Keywords:** cholesterol, obesity, hyperlipidemia, fatty acid, triglyceride, weight loss, [U-^13^C]palmitate, ALP, alkaline phosphatase, AST, aspartate transaminase, CBC, complete blood cell, CRTU, Clinical Research and Trials Unit, RBC, red blood cell, TG, triglyceride

## Abstract

Most research into red blood cell (RBC) lipids focuses on membrane phospholipids and their relationships to metabolic conditions and diet. Triglycerides (TGs) exist in most cells; the TG-fatty acids serve as readily available fuel for oxidative phosphorylation. Because RBCs lack mitochondria, they would not be expected to store fatty acids in TG. We followed up on a previous in vitro study that found FFA can be incorporated into RBC-TG by testing whether intravenously infused [U-^13^C]palmitate could be detected in RBC-TG. We also quantified RBC-TG fatty acid concentrations and profiles as they relate to plasma FFA and lipid concentrations. We found that *1*) RBC-TG concentrations measured by glycerol and LC/MS were correlated (*r* = 0.77; *P* < 0.001) and averaged <50 nmol/ml RBC; *2*) RBC-TG concentrations were stable over 18 h; *3*) [U-^13^C]palmitate was detectable in RBC-TG from half the participants; *4*) RBC-TGs were enriched in saturated fatty acids and depleted in unsaturated fatty acid compared with plasma FFA and previously reported RBC membrane phospholipids; *5*) RBC-TG fatty acid profiles differed significantly between obese and nonobese adults; *6*) weight loss altered the RBC-TG fatty acid profile in the obese group; and *7*) the RBC-TG fatty acid composition correlated with plasma lipid concentrations. This is the first report showing that plasma FFA contributes to RBC-TG in vivo, in humans, and that the RBC-TG fatty acid profile is related to metabolic health. The storage of saturated fatty acids in RBC-TG stands in stark contrast to the highly unsaturated profile reported in RBC membrane phospholipids.

The red blood cell (RBC) membrane consists of a lipid bilayer that contains cholesterol and phospholipids. The fatty acid composition of RBC membrane lipids may affect the functional characteristics of RBCs, which in turn can predispose to intravascular hemolysis and other diseases (1). The research on RBC lipids dates to the 1950s (2) and mainly focused on the incorporation of fatty acids into RBC membrane phospholipids using ^14^C-labeled FFA (3, 4, 5, 6). More recent research has focused on the RBC membrane lipid profile as it relates to metabolic conditions, including primary hyperlipidemia, obesity, gestational diabetes, and type 2 diabetes (7, 8, 9, 10, 11). The RBC membrane fatty acid composition is also measured as an indicator of dietary interventions for patients with metabolic conditions (12, 13, 14). This is because RBC membrane phospholipid fatty acids can be incorporated from the serum lipoproteins (15, 16). Factors such as diet and metabolic diseases can alter the fatty acid composition of the RBC membrane (17), which has a different fatty acid profile from plasma lipoprotein fatty acids (18, 19, 20). The RBC membrane phospholipids are enriched with unsaturated fatty acids (19), possibly related to the sphingomyelin content; sphingomyelin preferentially incorporates long-chain unsaturated fatty acids (21). However, RBCs have also been shown to have a small amount of triglyceride (TG) (4), which is unexpected because in many tissues intracellular TGs are precursors for fatty acid oxidation (22).

Mature RBCs are incapable of fatty acid oxidation because they lack mitochondria (15, 23), relying instead on glycolysis and pentose phosphate pathway for energy (24). Without the ability to oxidize fatty acids, it might be expected that all RBC fatty acids would be in the form of cell membrane phospholipids. Studies of RBCs done in the 1960s, however, suggest that nonesterified fatty acids can be incorporated into RBC-TG and RBC membrane phospholipids in vitro (4). Surprisingly, there has been very little research into RBC-TG since then, especially with regard to the source of fatty acids for RBC-TG and the fatty acid composition of this lipid pool.

We took advantage of ongoing studies of plasma FFA metabolism in humans to test the hypothesis that FFA can be incorporated into RBC-TG in vivo as well as to understand whether there are differences in the fatty acid profile(s) of RBC-TG between lean and obese humans. We confirmed that the presence of small, but measurable, RBC-TG pool documented that plasma FFA can be incorporated into RBC-TG and found remarkable differences in the fatty acid content of this pool compared with what is reported for RBC membrane phospholipids and plasma FFA. We also found that the RBC-TG fatty acid profile related to plasma lipid concentrations, obesity, and that it is modified by lifestyle-induced weight loss.

## Materials and methods

### Subjects

The samples to measure RBC-TG fatty acid and plasma FFA were obtained as part of a Mayo Clinic Institutional Review Board-approved research study designed to measure the effects of insulin on adipose tissue lipolysis in lean and obese volunteers as well as the effects of lifestyle-facilitated weight loss in the volunteers with obesity. The use of human material conforms to the principles outlined in the Declaration of Helsinki. The BMI range was 18.5–25 kg/m^2^ for the normal weight control group and 30–37 kg/m^2^ for the obese group. Participants were nonsmoking adults without diabetes, impaired fasting glucose, ischemic heart disease, atherosclerotic vascular disease, systemic inflammatory illness, thyroid disorders, and diseases or medications that alter FFA metabolism, adipose tissue metabolism, or metabolic rate. Female participants were premenopausal, and male participants were age matched to female participants. All volunteers were weight stable for at least 2 months prior to the study (±2% of body weight) and were provided an isoenergetic diet of consistent macronutrient distribution by the Mayo Clinic Clinical Research and Trials Unit (CRTU) for 3 days prior to the study visit.

During the screening visit, a blood sample was collected for measurement of a complete blood cell (CBC) count including the concentrations of hemoglobin, hematocrit, leukocyte (also called white blood cell), platelet, as well as a lipid panel—fasting plasma TG, total cholesterol, HDL-C, and LDL-C. We also measured fasting plasma glucose concentrations, aspartate transaminase (AST), alkaline phosphatase (ALP), and creatinine. Each volunteer had body composition measures using dual-energy X-ray absorptiometry (25).

### Study design

The evening prior to studies, the participants were admitted to the Mayo Clinic CRTU at approximately 1700 h. The next morning, an intravenous catheter was placed in a retrograde fashion in the hand for blood sampling, and another was placed in a forearm vein for palmitate tracer, hormone, and glucose infusions. Arterialized venous blood samples were collected using the hot box technique with a temperature of 55°C (26) and immediately placed in tubes containing EDTA. At 0700 h, an infusion of [U-^13^C]palmitate (∼300 nmol/min) was initiated to trace palmitate kinetics and to test for incorporation of plasma palmitate into RBC-TG. This infusion was continued until the completion of the study. Blood samples were collected at 10 min intervals between 0830 and 0900 h to measure plasma palmitate concentration and enrichment. The participants then underwent a pancreatic (somatostatin)-insulin clamp study with three sequential, 2 h insulin infusion rates (0, 0.25, and 1 mU·kg^−1^·min^−1^) together with an infusion of 50% dextrose to maintain blood glucose concentrations at ∼5.0 mmol/l; the dextrose infusion rate was adjusted as needed by measuring plasma glucose concentrations every 10 min. Blood samples for RBC-TG fatty acid concentrations were collected at the completion of the insulin clamp study (before the [U-^13^C]palmitate infusion was stopped) and again the next morning in the fasting state (18 h after the previous blood sample for RBC-TG).

The obese group underwent a comprehensive lifestyle intervention with the goal of achieving a weight loss of ∼10%, following which they were admitted to the CRTU, where they underwent a second study identical to the first study.

### Methods

#### RBC sample wash

The blood samples from each study were immediately placed on ice and then centrifuged to collect the plasma and RBC fractions. The RBC fraction was washed three times using isotonic saline (four times the RBC volume) to remove all plasma. Conical tubes used for the first two washes were centrifuged at 1,000 *g* for 10 min at 10°C for each wash (J-20 XPI; Beckman Coulter, Pasadena, CA). Flat base screw cap tubes were used for the third wash and centrifuged at 1,500 *g* for 15 min at 10°C. The supernatant was removed after each wash. The RBC samples from each volunteer were stored at −80°C until the samples were analyzed.

#### RBC-TG extraction

In order to account for possible incomplete extraction of RBC-TGs, we added ∼11,000 dpm of [1-^14^C]triolein (PerkinElmer, Boston, MA; catalog number: NEC-674) to 1 ml of each RBC sample that had been suspended in 5 ml of methanol and 10 ml of chloroform. The samples were allowed to sit in cold room for 48 h, following which we added 3.75 ml of 0.88% potassium chloride. After 20 min, we vortexed the samples and then centrifuged them for 10 min at 2,000 rpm. The lower phase containing the lipids was collected, another 10 ml of chloroform was added to the RBC fraction, and the sample was vortexed and centrifuged again. The lower phase again was collected, combined with the first lower phase sample, and dried down. The RBC-TG was isolated from other lipids using an SPE column (LC-Si tubes; Supelclean, PA) that had been prepared with 3 ml of hexane; the RBC lipid extracts were suspended in hexane. In order to fully load the RBC lipids onto the columns, the samples were loaded first using 3 ml hexane, followed by a second and third aliquot of 2 ml each. Hexane, ether, and methylene chloride were mixed with the proportion of 89:1:10, and the samples were washed with 15 ml of the mixture solvent to elute the RBC-TG fraction.

A fraction of the RBC-TG fatty acid sample was analyzed using a scintillation counter (PerkinElmer, Inc, Waltham, MA) to calculate the recovery of RBC-TG fatty acids. Another portion of the RBC-TG fatty acid fraction was processed for analysis using LC/MS to determine fatty acid concentrations and palmitate enrichment from the [U-^13^C]palmitate (27). The RBC-TG concentrations were measured using the glycerol concentrations measured in the infranatant using a COBAS INTEGRA 400 plus and the Roche (Indianapolis, IN) TRIGL kit (28) corrected for the fractional recovery of ^14^C-oleate from the ^14^C-triolein.

To measure the individual RBC-TG fatty acid concentrations and palmitate enrichment, the RBC-TGs were hydrolyzed with 1 ml of a 5 μl of 10 N NaOH/ml in methanol and heptane (8:2) incubated for 30 min at 60°C. The resultant fatty acid fraction was dried down and then extracted using the Dole method (29) (100 μl of 0.01 M KPO_4_ buffer, 5 ml of isopropyl alcohol:heptane:1 N H_2_SO_4_—40:10:1). To create the concentration standard curve, we use serial dilutions of a mixed fatty acid standard solution with 12 fatty acid species (palmitic acid, oleic acid, linoleic acid, palmitoleic acid, palmitelaidic acid, elaidic acid, linolenic acid, arachidonic acid, myristic acid, stearic acid, EPA, and DHA) with a range of 10 different concentrations; we use two different quality control samples with each assay to assure that interassay variability is minimal. The mixed fatty acid concentration standard solution was prepared using pure (as measured by LC/MS), gravimetrically measured fatty acids (Sigma-Aldrich, St. Louis, MO) suspended in fatty acid-free bovine serum albumin (Sigma-Aldrich). The amounts of each fatty acid we include are proportional to their usual presence in human plasma. We added 80 ng of C17-fatty acid internal standard (Fluka; Sigma-Aldrich; catalog number: 51610) to each sample and simultaneously to a fatty acid standard curve that included the following species. Each sample was shaken for 90 min after which 2 ml heptane and 3 ml H_2_O were added, followed by centrifugation for 10 min at 2,000 rpm. The upper phase was collected as the RBC-TG fatty acid sample, and the lower phase containing glycerol was neutralized with 5 μl of 10 N NaOH, dried down, and resuspended in 0.4 ml of water for measurement of total RBC-TG (see aforementioned details). The samples are analyzed using an Acquity UPLC system (Waters Corporation, Milford, MA) coupled to an Agilent 6120 (single quadrupole) LC/MS (Agilent Technologies, Lexington, MA) for both concentration and enrichment (27).

### Statistical analysis

We used a *P* value of less than 0.05 as our test for statistical significance for a priori hypotheses and a *P* value of less than 0.01 for post hoc analyses. To test for between-group differences in subject characteristics, plasma lipid, and CBC concentrations, we used nonpaired *t*-tests if data were normally distributed. If the data were not normally distributed, it was log transformed before analysis. If the data could not be transformed to achieve a normal distribution, Wilcoxon signed-rank test was performed. To test the differences in the distribution of men and women between groups, we used Chi-square tests, and to test for differences between before weight loss and after weight loss visits of the obese group, paired *t*-tests were performed if data were normally distributed and on log-transformed data if the distribution was not normal. Correlations between the clinical characteristics and plasma FFA or RBC-TG fatty acid were performed using Pearson test if the data were normally distributed or could be transformed to achieve a normal distribution. The Spearman rank test was applied if otherwise. Statistical analysis was performed using SPSS, version 20.0 (SPSS, Inc, Chicago, IL).

## Results

### Subject characteristics

A total of 25 volunteers participated in this study (Table 1). The normal weight group (n = 12) had one study visit, and 11 of the 13 participants in the obese group underwent studies before and after weight loss (two volunteers in the obese group were unable to lose enough weight to be eligible for the repeat study). Plasma lipid concentrations were not measured on the blood samples from one postweight loss study. Twenty-three participants were Caucasian, one was Hispanic, and one self-designated as “other ethnicity.” The distribution of sex and age was similar in the normal weight and obese groups. Fasting plasma TG concentrations were greater in the obese than normal weight group, whereas HDL-C was greater in the normal weight group. Total cholesterol, LDL-C, and non-HDL-C decreased significantly in the obese group after weight loss. The CBC, AST, ALP, and creatinine were only measured in the beginning of the study; the AST, ALP, and creatinine were not different between normal weight and obese groups. The normal weight group had lower white blood cell than the obese group. As expected, the obese group had more body fat than the normal weight group.

**Table 1:** Participant characteristics in each group and study visit

Parameter	Obese				Lean	
	Previsit		Postvisit			
	n		n		n	
Sex (female/male)	13	5/8	11	5/6	12	6/6
Age (years)	13	40 ± 11	11	41 ± 10	12	40 ± 11
BMI (kg/m^2^)	13	34.6 ± 1.8	11	30.6 ± 2.5^a^	12	23.6 ± 1.4^b^
Hematology measurements						
Total cholesterol (mg/dl)	13	180 ± 19	10	157 ± 10^a^	12	176 ± 23
TG (mg/dl)	13	103 ± 39	10	81 ± 19	12	71 ± 30^c^
HDL-C (mg/dl)	13	51 ± 12	10	46 ± 10	12	69 ± 16^d^
LDL-C (mg/dl)	13	108 ± 20	10	94 ± 13^e^	12	93 ± 26
Hemoglobin (g/dl)	13	14.3 ± 1.0	N/A		12	14.2 ± 1.1
Hematocrit (%)	13	43.1 ± 2.0	N/A		12	42.5 ± 2.8
Leukocyte (×10^9^/l)	13	6.4 ± 1.2	N/A		12	4.9 ± 0.8^d^
Platelet (×10^9^/l)	13	259 ± 44	N/A		12	228 ± 37
Fasting plasma glucose (mg/dl)	13	90 ± 11	N/A		12	88 ± 5
Body fat (%)	13	41 ± 6	11	37 ± 6^a^	12	28 ± 8^b^

Data was presented as mean value ± SD.

a *P* < 0.001 versus previsit of obese group.

b *P* < 0.001 versus obese group.

c *P* < 0.05 versus obese group.

d *P* < 0.01 versus obese group.

e *P* < 0.01 versus previsit of obese group.

### RBC-TG fatty acid concentrations

**Fig. 1:**
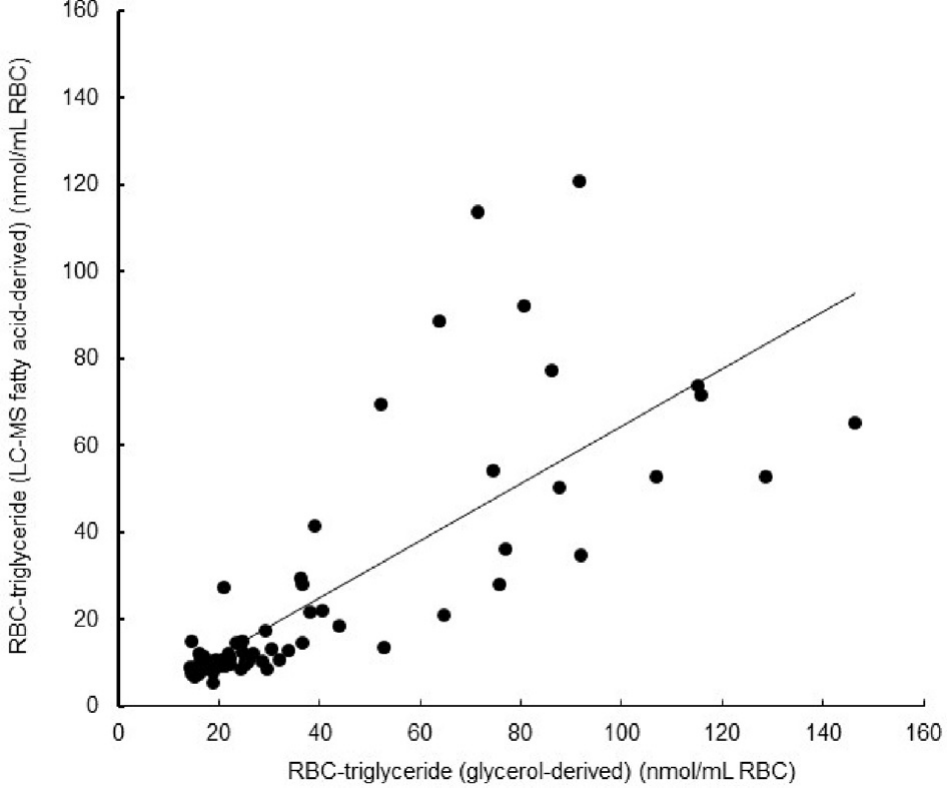
Concentrations of RBC-TG fatty acid determined by glycerol versus LC-MS (nmol/ml RBC, n = 65) were correlated (*r* = 0.77; *P* < 0.001). The mean ± SD RBC-TG concentrations were 28 ± 28 and 42 ± 33 nmol/ml as measured by LC-MS and glycerol-based methods, respectively (*P* < 0.001).

The concentration of RBC-TG (nmol/1 ml of RBC) was measured using the glycerol content of the RBC-TG eluate from the column, and the sum of the concentrations of the 12 RBC-TG fatty acid species was measured by LC/MS ÷ 3; both approaches used the recovery factor from the ^14^C-triolein. Because of technical problems with four of the samples, we were unable to measure total RBC-TG concentrations using both methods, thus the comparison between the two approaches was carried using data from 65 samples. The concentrations calculated from the two approaches were correlated (*r* = 0.77; *P* < 0.001; Fig. 1), but the concentrations measured using the glycerol method were greater than those measured using LC/MS (42 ± 32 vs. 28 ± 28 nmol/ml RBC, respectively; *P* < 0.001).

We compared the RBC-TG fatty acid concentrations from blood samples drawn 18 h apart in order to assess intraindividual variability of this parameter. Blood samples from both time points were not available for three participants; therefore, the analysis was carried out using 22 pairs of samples. The concentrations and contribution of the different species of fatty acids to RBC-TG were not different between the two time points (Table 2).

**Table 2:** RBC-TG fatty acid compositions and concentrations at two time points (n = 22)

Fatty Acid Species	Composition (%)		Concentration (nmol/ml RBC)	
	Afternoon	18 h Later	Afternoon	18 h Later
Saturated fatty acids				
Myristic (14:0)	12.6 ± 6.4	13.0 ± 8.1	1.2 ± 0.8	1.4 ± 1.3
Palmitic (16:0)	39.9 ± 8.6	36.8 ± 5.8	3.7 ± 2.1	3.8 ± 2.5
Stearic (18:0)	14.7 ± 6.6	13.4 ± 5.8	1.4 ± 1.1	1.5 ± 1.4
Monounsaturated fatty acids				
Palmitoleic + palmitelaidic (16:1 n-7 + 16:1 trans-9)	6.7 ± 5.0	6.4 ± 3.3	0.6 ± 0.7	0.5 ± 0.3
Oleic (18:1 n-9)	13.8 ± 8.3	15.6 ± 8.6	1.3 ± 1.1	1.5 ± 1.2
Elaidic (18:1 trans-9)	3.5 ± 2.9	3.5 ± 2.4	0.4 ± 0.6	0.4 ± 0.5
Polyunsaturated ω-3 fatty acids				
EPA (20:5 n-3)	1.7 ± 2.6	2.0 ± 3.3	0.2 ± 0.4	0.2 ± 0.4
DHA (22:5 n-3)	1.9 ± 1.3	2.5 ± 1.5	0.2 ± 0.1	0.1 ± 0.1
Polyunsaturated ω-6 fatty acids				
Linoleic (18:2 n-6)	3.7 ± 2.1	4.8 ± 3.0	0.4 ± 0.4	0.5 ± 0.6
Linolenic (18:3 n-6)	0.2 ± 0.1	0.2 ± 0.1	0.02 ± 0.02	0.02 ± 0.01
Arachidonic (20:4 n-6)	1.2 ± 0.7	1.5 ± 0.9	0.1 ± 0.1	0.1 ± 0.1
Total fatty acid concentration (nmol/ml)	NA		9.4 ± 4.7	10.2 ± 5.9

NA, not available.

Data presented as mean value ± SD.

The individual plasma FFA concentrations were measured before the start of the experiments on the study day for comparison with the individual RBC-TG fatty acid concentrations and percent composition (Table 3). There were major significant differences in the percent of fatty acids in RBC-TG compared with plasma FFA. The RBC-TGs were enriched in saturated fatty acids compared with plasma and were depleted in 18-carbon unsaturated fatty acids (except elaidic).

**Table 3:** RBC-TG fatty acid versus plasma FFA compositions and concentrations (n = 25)

Fatty Acid Species	RBC-TG Fatty Acid	Plasma FFA	RBC TG-FA Concentration	Plasma FFA Concentration
	Composition (%)		(nmol/ml RBC)	(nmol/mL)
Saturated fatty acids				
Myristic (14:0)	13.4 ± 7.3	2.2 ± 0.3^a^	1.2 ± 1.0	11 ± 5^a^
Palmitic (16:0)	38.2 ± 5.7	24.0 ± 2.6^a^	3.5 ± 2.0	115 ± 39^a^
Stearic (18:0)	14.1 ± 5.1	8.2 ± 2.1^a^	1.3 ± 1.1	37 ± 8^a^
Monounsaturated fatty acids				
Palmitoleic + palmitelaidic (16:1 n-7 + 16:1 trans-9)	6.8 ± 3.9	2.8 ± 1.4^a^	0.6 ± 0.4	14 ± 8^a^
Oleic (18:1 n-9)	14.1 ± 7.6	33.8 ± 3.7^a^	1.3 ± 1.0	161 ± 59^a^
Elaidic (18:1 trans-9)	3.3 ± 2.2	1.9 ± 0.6^b^	0.4 ± 0.4	9 ± 4^a^
Polyunsaturated ω-3 fatty acids				
EPA (20:5 n-3)	1.9 ± 2.1	0.5 ± 0.2^b^	0.2 ± 0.3	2 ± 1^a^
DHA (22:5 n-3)	2.4 ± 1.4	1.0 ± 0.9^a^	0.2 ± 0.1	5 ± 6^a^
Polyunsaturated ω-6 fatty acids				
Linoleic (18:2 n-6)	4.2 ± 2.2	22.2 ± 2.0^a^	0.4 ± 0.4	108 ± 41^a^
Linolenic (18:3 n-6)	0.2 ± 0.1	2.4 ± 0.4^a^	0.02 ± 0.01	12 ± 6^a^
Arachidonic (20:4 n-6)	1.4 ± 0.7	1.1 ± 0.3^c^	0.1 ± 0.1	5 ± 3^a^
Total fatty acid concentration (nmol/ml)	NA		9 ± 5	479 ± 161^a^

NA, not available.

Data are presented as mean value ± SD. The plasma FFA concentrations were those measured in the overnight at the postabsorptive state, at the day of the study.

a *P* < 0.001 versus RBC-TG fatty acid composition/concentration.

b *P* < 0.01 versus RBC-TG fatty acid composition.

c *P* < 0.05 versus RBC-TG fatty acid composition.

### Incorporation of plasma FFA-[U-^13^C]palmitate into RBC-TG-palmitate

We tested for the presence of [U-^13^C]palmitate in RBC-TG-palmitate using LC/MS; its presence would provide evidence that plasma FFA-palmitate was taken up by RBCs and stored using the TG synthesis pathway. We found clearly detectable enrichment in 38 of 69 RBC-TG-palmitate samples. The average plasma FFA-palmitate enrichment during the study was greater than the RBC-TG-palmitate enrichment (0.709 ± 0.349 vs. 0.088 ± 0.120, respectively; *P* < 0.0001). For samples from the same individual collected at the completion of the [U-^13^C]palmitate infusion and 18 h later, we found no significant change in palmitate enrichment. This indicates that, once stored, plasma FFA-palmitate does not rapidly exit the RBC-TG fatty acid pool.

The samples with no detectable RBC-TG palmitic enrichment did not differ from samples with detectable enrichment in terms of whether the participant was lean or obese, RBC-TG palmitate concentrations, plasma palmitate concentrations, the ratio of plasma FFA-palmitate to RBC-TG palmitate concentrations, or plasma palmitate enrichment.

### Effects of obesity and weight loss on RBC-TG fatty acids and plasma FFA

The RBC-TG fatty acid and fasting plasma FFA concentrations in the normal weight and obese groups are provided in Table 4. There was not a significant difference in the total RBC-TG fatty acid concentrations or fasting plasma FFA concentrations between normal weight and obese volunteers. There were a few between-group differences in the contributions of the minor fatty acid species to total plasma FFA, and there were significant differences in the contribution of major fatty acid species to RBC-TG fatty acid content. The RBC-TG fatty acids from obese volunteers were enriched in palmitate and stearate compared with normal weight volunteers, whereas the RBC-TG fatty acids from normal weight volunteers were enriched in oleate, palmitoleate, and palmitelaidic acid.

**Table 4:** RBC-TG fatty acid and plasma FFA composition and concentrations of obese (n = 13) versus lean (n = 12) groups

Fatty Acid Species	RBC TG-FA		Plasma FFA		RBC TG-FA Concentration		Plasma FFA Concentration	
	Composition (%)				(nmol/ml RBC)		(nmol/mL)	
	Obese	Lean	Obese	Lean	Obese	Lean	Obese	Lean
Saturated fatty acids								
Myristic (14:0)	16.1 ± 7.7	10.5 ± 5.9	2.4 ± 0.4	2.1 ± 0.3^a^	1.3 ± 0.7	0.9 ± 0.8	11 ± 4	10 ± 4
Palmitic (16:0)	40.5 ± 5.9	35.8 ± 4.3^b^	24.6 ± 1.4	23.3 ± 3.5	3.9 ± 2.4	2.9 ± 1.0	108 ± 31	110 ± 32
Stearic (18:0)	16.9 ± 5.3	11.1 ± 2.8^c^	8.6 ± 2.0	7.7 ± 2.2	1.7 ± 1.3	0.9 ± 0.4	36 ± 5	35 ± 10
Monounsaturated fatty acids								
Palmitoleic + palmitelaidic (16:1 n-7 + 16:1 trans-9)	5.4 ± 3.9	8.3 ± 3.6^b^	3.1 ± 1.1	2.4 ± 1.5	0.5 ± 0.3	0.7 ± 0.4	14 ± 6	12 ± 10
Oleic (18:1 n-9)	9.0 ± 4.2	19.6 ± 6.6^d^	32.7 ± 2.2	35.0 ± 4.6	0.8 ± 0.4	1.7 ± 1.2^b^	145 ± 45	168 ± 63
Elaidic (18:1 trans-9)	2.6 ± 2.8	4.0 ± 1.1^c^	2.1 ± 0.6	1.6 ± 0.5^a^	0.3 ± 0.6	0.4 ± 0.2	10 ± 4	8 ± 4
Polyunsaturated ω-3 fatty acids								
EPA (20:5 n-3)	2.6 ± 2.6	1.2 ± 0.8	0.4 ± 0.2	0.5 ± 0.3	0.3 ± 0.4	0.1 ± 0.1	2 ± 1	2 ± 2
DHA (22:5 n-3)	1.8 ± 1.0	3.0 ± 1.5	0.6 ± 0.3	1.5 ± 1.0^e^	0.1 ± 0.7	0.2 ± 0.1^b^	3 ± 3	7 ± 4^a^
Polyunsaturated ω-6 fatty acids								
Linoleic (18:2 n-6)	3.7 ± 1.7	4.6 ± 2.6	22.0 ± 2.0	22.4 ± 0.2	0.3 ± 0.2	0.5 ± 0.5	98 ± 31	106 ± 34
Linolenic (18:3 n-6)	0.2 ± 0.1	0.2 ± 0.1	2.5 ± 0.3	2.3 ± 0.5	0.02 ± 0.01	0.02 ± 0.01	11 ± 4	11 ± 6
Arachidonic (20:4 n-6)	1.1 ± 0.6	1.7 ± 0.6	1.0 ± 0.2	1.2 ± 0.4	0.09 ± 0.06	0.15 ± 0.11	4 ± 1	6 ± 3
Total fatty acid concentration (nmol/ml)	NA				9.3 ± 4.7	8.5 ± 3.7	442 ± 123	475 ± 145

NA, not available.

Data were presented as mean value ± SD.

a *P* < 0.05 versus plasma FFA composition/concentration of obese group.

b *P* < 0.05 versus RBC-TG fatty acid composition/concentration of obese group.

c *P* < 0.01 versus RBC-TG fatty acid composition/concentration of obese group.

d *P* < 0.001 versus RBC-TG fatty acid composition/concentration of obese group.

e *P* < 0.01 versus plasma FFA composition/concentration of obese group.

The RBC-TG fatty acid and fasting plasma FFA concentrations were measured using samples from the same time points before weight loss and after weight loss for the obese volunteers. The total RBC-TG fatty acid concentrations decreased significantly (Table 5). This was largely driven by a decrease in the content of saturated fatty acids, whereas the contribution of monounsaturated fatty acid increased (Table 5). We also found changes in the percent of the plasma FFA species to total FFA concentrations (Table 5), mostly related to changes in the contribution of oleic acid to total FFA.

**Table 5:** RBC-TG fatty acid and plasma FFA compositions and concentrations of before weight loss versus after weight loss visits (obese group, n = 11)

Fatty Acid Species	RBC-TG Fatty Acid		Plasma FFA		RBC-TG Fatty Acid Concentration		Plasma FFA Concentration	
	Compositions (%)				(nmol/ml RBC)		(nmol/mL)	
	Previsit	Postvisit	Previsit	Postvisit	Previsit	Postvisit	Previsit	Postvisit
Saturated fatty acids								
Myristic (14:0)	17.6 ± 7.4	7.3 ± 1.1^a^	2.4 ± 0.4	2.0 ± 0.3^b^	1.4 ± 0.7	0.4 ± 0.1^a^	10 ± 3	12 ± 3
Palmitic (16:0)	40.0 ± 6.3	37.6 ± 3.2	24.5 ± 1.5	25.0 ± 3.8	3.5 ± 2.0	2.2 ± 0.4^c^	105 ± 19	146 ± 33^b^
Stearic (18:0)	17.0 ± 5.3	12.1 ± 1.5^c^	8.9 ± 1.9	7.0 ± 1.9^d^	1.6 ± 1.1	0.7 ± 0.1^c^	37 ± 5	40 ± 8
Monounsaturated fatty acids								
Palmitoleic + palmitelaidic (16:1 n-7 +16:1 trans-9)	5.6 ± 4.1	6.3 ± 1.2	3.1 ± 1.2	1.8 ± 0.5^b^	0.5 ± 0.4	0.4 ± 0.1	13 ± 6	11 ± 5
Oleic (18:1 n-9)	8.3 ± 3.0	21.7 ± 3.3^e^	32.5 ± 2.2	39.1 ± 3.7^f^	0.7 ± 0.3	1.3 ± 0.4^a^	140 ± 29	236 ± 81^b^
Elaidic (18:1 trans-9)	1.8 ± 1.6	3.9 ± 0.8^a^	2.2 ± 0.6	1.7 ± 0.6^d^	0.2 ± 0.2	0.2 ± 0.04	10 ± 4	10 ± 4
Polyunsaturated ω-3 fatty acids								
EPA (20:5 n-3)	2.5 ± 2.7	1.0 ± 0.4	0.5 ± 0.2	0.3 ± 0.1^d^	0.2 ± 0.3	0.1 ± 0.02	2 ± 1	2 ± 1
DHA (22:5 n-3)	1.9 ± 1.0	3.1 ± 1.0^c^	0.5 ± 0.1	1.4 ± 0.9^b^	0.1 ± 0.1	0.2 ± 0.1	2 ± 1	8 ± 4^f^
Polyunsaturated ω-6 fatty acids								
Linoleic (18:2 n-6)	4.0 ± 1.7	4.9 ± 1.3	21.9 ± 2.2	18.2 ± 9.2	0.3 ± 0.2	0.3 ± 0.1	95 ± 24	120 ± 82
Linolenic (18:3 n-6)	0.2 ± 0.1	0.2 ± 0.1	2.4 ± 0.3	2.3 ± 0.5	0.02 ± 0.01	0.01 ± 0.01	11 ± 3	14 ± 6
Arachidonic (20:4 n-6)	1.1 ± 0.6	2.1 ± 0.8^c^	1.0 ± 0.2	1.0 ± 0.2	0.1 ± 0.1	0.1 ± 0.04	4 ± 1	6 ± 3
Total fatty acid concentration (nmol/ml)	NA				8.6 ± 3.6	5.8 ± 0.9^c^	429 ± 84	606 ± 201^d^

NA, not available.

Data are presented as mean value ± SD.

a *P* < 0.01 versus RBC-TG fatty acid composition/concentration of before weight loss visit.

b *P* < 0.01 versus plasma FFA composition/concentration of before weight loss visit.

c *P* < 0.05 versus RBC-TG fatty acid composition/concentration of before weight loss visit.

d *P* < 0.05 versus plasma FFA composition/concentration of before weight loss visit.

e *P* < 0.001 versus RBC-TG fatty acid composition/concentration of before weight loss visit.

f *P* < 0.001 versus plasma FFA composition/concentration of before weight loss visit.

### Relationships between RBC-TG fatty acid composition and plasma lipid concentrations

Because previous studies have shown that RBC membrane phospholipid fatty acid content is related to plasma lipid concentrations, we performed post hoc, exploratory analyses to test whether the same is true for RBC-TG fatty acid content. To do this, we used the average and baseline RBC-TG fatty acid concentrations and composition from the duplicate samples from all volunteers to test whether they were related to plasma lipid concentrations. The only associations that met our criteria for statistical significance (*P* < 0.01) were a negative correlation between plasma TG concentrations and RBC-TG arachidonate (*r* = −0.53; *P* < 0.01) and a positive correlation between HDL-C concentrations and RBC-TG palmitoleate (*r* = 0.57; *P* < 0.01). These seem unlikely to be chance associations because there were also associations between fasting plasma TG concentration and RBC-TG content of stearate (*r* = 0.46; *P* < 0.05), linoleate (*r* = −0.41; *P* < 0.05), linolenate (*r* = −0.44; *P* < 0.05), DHA (*r* = −0.45; *P* < 0.05), palmitoleate (*r* = −0.49; *P* < 0.05), and palmitelaidic (*r* = −0.47; *P* < 0.05) that did not meet our criteria of *P* < 0.01 for post hoc testing. In addition, HDL-C concentrations were associated with RBC-TG oleate (*r* = 0.50; *P* < 0.05), arachidonate (*r* = 0.42; *P* < 0.05), DHA (*r* = 0.40; *P* < 0.05), palmitate (*r* = −0.51; *P* < 0.05), and stearate (*r* = −0.50; *P* < 0.05); again these did not meet our criteria for post hoc testing but displayed a pattern of consistency that we found difficult to dismiss.

## Discussion

This study was designed to investigate the characteristics of RBC-TG fatty acids and whether they can be derived from plasma FFA in vivo. We measured the concentration and composition of RBC-TG fatty acids and examined whether they are altered in adults with obesity, whether they change with weight loss, and whether they are related to plasma lipid concentrations. We found low, but easily detectable, concentrations of RBC-TG fatty acids and documented the incorporation of plasma FFA into RBC-TG using stable isotope tracer approaches. Adults with obesity had an RBC-TG fatty acid profile that was enriched with saturated fatty acids compared with nonobese and the pattern changed with weight loss. The relative contributions of many saturated fatty acids to RBC-TG were positively correlated with plasma lipid concentrations but negatively correlated with HDL-C concentrations. Our study provides strong evidence that plasma FFAs are directly incorporated into RBC-TG, that this process favors the presence of saturated fatty acids, and that obesity/lipid disorders are related to the contribution of different fatty acid species in RBC-TG. Furthermore, weight loss and its improved metabolic status changes RBC-TG fatty acid profiles.

The concentrations and composition of RBC-TG fatty acids we found are significantly different than the RBC membrane phospholipid fatty acids that have been widely reported (8, 30, 31, 32). The concentration of RBC membrane phospholipid fatty acids was reported to average approximately 14.3 μmol/ml RBC (8), 500 times greater than the average RBC-TG fatty acid concentrations we found. This difference in concentrations between RBC membrane phospholipid and RBC-TG fatty acids is consistent with the report of de Gómez Dumm *et al.* (30), who found RBC-phospholipid content was 340 times greater than RBC-TG in membranes. With regard to the distribution of fatty acid species, we found that RBC-TG had greater proportions of 14- and 16-carbon saturated fatty acids, as well as 16:1 fatty acid, compared with what has been reported for RBC phospholipids (8). The relative contributions of 18:0 and 18:1 fatty acids in RBC-TG were similar to those reported for RBC phospholipids, but RBC-TG had less linoleate and arachidonate than that reported for RBC-membrane fatty acids (20, 31, 32). Among adults with BMIs similar to those of our volunteers, RBC membrane total fatty acid or phospholipids contained more linolenate and arachidonate but less palmitate (33, 34) than we found in RBC-TG. Thus, although plasma FFA can be incorporated into both RBC membrane lipids (3, 4, 5, 6) and RBC-TG, there is clearly favoritism when it comes to the pool in which the different species are stored.

Several investigators have reported that there are differences between obese and lean humans in RBC membrane fatty acid profiles (35, 36). Some found that, compared with obese humans, RBC membrane phospholipids from lean humans contain more arachidonate and DHA but less linoleate (35), whereas others found the opposite pattern (36). Weight loss reduces the contribution of palmitate and increases the content of arachidonate to RBC membrane fatty acids (37), similar to what we observed for the RBC-TG pool after weight loss. Treatment of hyperlipidemic patients with lifestyle or medication reduced the stearate contribution in RBC membrane phospholipids (13, 14), whereas arachidonate (13), oleate, and DHA (14) increased. These changes are similar to those we observed for the RBC-TG fatty acid profile in response to weight loss. Thus, although RBC membrane and RBC-TG fatty acids have different profiles, they respond to weight loss or lifestyle intervention in somewhat similar fashions. Whether RBCs have cellular functions that discriminate toward different fatty acids in response to these interventions or whether they are passive respondents to a changing fatty acid milieu does not appear to be known. However, the positive correlations between RBC-TG fatty acid profile and plasma lipid concentrations indicate that there might be regulation of how RBC allocates plasma FFA pool toward the RBC-TG fatty acid that changes in concert with metabolic conditions.

RBC membrane fluidity is greater when the content of unsaturated fatty acids is greater; increased palmitate content of RBC membrane reduces RBC membrane fluidity (38). For example, fish oil supplements that increased RBC membrane DHA and EPA improved RBC membrane fluidity (39). It seems unlikely that RBC can be selective about the species of plasma FFA they take up. Thus, it is possible that RBC-TGs, which are clearly not used as an energy source, may serve as a benign way to sequester some saturated fatty acids, whereas polyunsaturated fatty acids are shunted to the RBC membrane phospholipids to optimize membrane fluidity. However, further research will be needed to test this hypothesis as well as to understand how this selectivity process is regulated.

The amount of plasma FFA-palmitate stored in RBC-TG was not large. There was no detectable [U-^13^C]palmitate in half of the RBC samples, and the RBC-TG palmitate enrichment in other half averaged only ∼10% of the plasma palmitate enrichment. Those observations, combined with the fact that the RBC-TG fatty acid concentrations, composition, and RBC-TG-palmitate enrichment were stable over 18 h, suggest that this small RBC lipid pool turns over slowly. Studies of RBC membrane phospholipid fatty acid turnover using DHA and EPA supplements suggest that the half-life of RBC membrane DHA and EPA was 2–4 weeks (40, 41), also a relatively slow turnover rate. Given that RBCs have a lesser demand for fatty acid utilization, it is perhaps unsurprising that the turnover of fatty acids in the various pools is less than more metabolically active tissues (42, 43). In the future, a longer continuous infusion of stable isotope-labeled FFA under constant postabsorptive conditions could help better define this process. In addition, more sophisticated studies of RBC metabolism might be needed to help understand whether RBC characteristics themselves determine whether/how much plasma FFA is incorporated into RBC-TG.

We measured the RBC-TG concentrations using two approaches: a glycerol-based assay of the TG fraction from RBC lipid extract and the sum of the individual fatty acid concentrations from that same fraction measure by LC/MS. The TG concentrations derived using the LC/MS approach were well correlated with, but less than, those obtained using the glycerol approach. We suggest that this is because our standard assay measures the 12 major fatty acid species found in plasma, and there are no doubt regarding the additional fatty acid species in RBC-TG. From previous investigations of RBC membrane fatty acid species, the 12 fatty acids we measured by LC/MS accounted for 64–80% of total RBC membrane fatty acids (8, 10, 31). If the same is true for RBC-TG, this could explain the difference between the two approaches.

As with all studies, there are some limitations to this one. The infusion of [U-^13^C]palmitate was partially under hyperinsulinemic conditions, which suppress FFA concentrations. It may be that the modest incorporation of the palmitate tracer into RBC-TG was somewhat limited to the extent that lower FFA concentrations limit FFA incorporation into RBC-TG and that we underestimated the kinetics of this process. In addition, we did not monitor the RBC-TG-palmitate enrichment over a long enough time interval to estimate the turnover of RBC-TG. Furthermore, because there may be different turnover rates of different fatty acid species, future studies should consider using tracers of multiple FFA species. This is an advantage of stable isotopes in that multiple tracers can be employed. Unfortunately, we did not measure the incorporation of plasma palmitate into the other RBC membrane lipids and therefore cannot address the question of incorporation of plasma FFA-palmitate into other RBC lipid pools.

## Conclusion

The current study is the first to confirm that plasma FFAs are incorporated into RBC-TG fatty acids in vivo, in humans. We also are the first to describe the relationships between RBC-TG fatty acid profiles and serum lipids as well as how obesity and weight loss affect the RBC-TG fatty acid profiles. Although very small, the RBC-TG pool clearly interacts with plasma fatty acids and may serve as a place to sequester fatty acid species that, if incorporated into membrane phospholipids, would reduce RBC membrane fluidity. These findings suggest that RBCs are surprisingly sophisticated when it comes to differential partitioning of fatty acids derived from the circulation. Further investigation into RBC-TG fatty acid metabolism could lead to a better understanding of RBC abnormalities in metabolic disorders.

## Data availability

Data described previously are contained within the article. Data sharing is available to this article upon reasonable request. Contact information of data sharing: Michael D Jensen, jensen@mayo.edu.

## Conflict of interest

The authors declare that they have no known conflicts of interests or personal relationships that could have appeared to influence the work reported in this article.
